# Palliative Care education in Armenia: perspectives of first-year Armenian physician residents

**DOI:** 10.1186/s12904-022-00938-z

**Published:** 2022-04-20

**Authors:** Carolin Hagedorn, Artashes Tadevosyan, Stephen Mason, Frank Elsner

**Affiliations:** 1 Department of Palliative Medicine, RWTH Aachen University, Aachen, Germany; 2 Department of Public Health and Health Care, Yerevan State Medical University, Yerevan, Armenia; 3 Palliative Care Unit, University of Liverpool, Liverpool, UK

**Keywords:** Armenia, Palliative Care, Education, Qualitative research, Interview, Undergraduate medical education, Global Health

## Abstract

**Background:**

Due to developing demographic changes, including an aging society and the increasing prevalence of non-communicable diseases, Palliative Care is increasingly highlighted as a universal healthcare need. The need for Palliative Care in Armenia is set against the context of an underdeveloped healthcare system. Further, the absence of palliative medicine within medical education, particularly undergraduate education in Armenia presents a major barrier to improving care. This research aimed to assess the perception of young Armenian physicians’ understanding of Palliative Care, its perceived status in Armenia and the experience and influence of any engaged Palliative Care education.

**Methods:**

Twenty Armenian first-year residents with different specializations were interviewed July and September 2016 regarding: understanding/knowledge, experiences, perceived competence, and expectations of Palliative Care and Palliative Care education. The transcripts from these semi-structured interviews were analyzed using Qualitative Content Analysis.

**Results:**

Participants perceived that Armenia’s health care system lacked sufficient Palliative Care and Palliative Care education. Although elements of Palliative Care were included in different specialty teaching, this provided just a partial understanding of typical Palliative Care patients/symptoms, approaches to holistic care, and crucially key communication skills. Challenges noted by participants in caring for Palliative Care patients included emotional difficulties, communication of diagnosis/prognosis, uninformed patients and concerns for patients, families, and physicians. Self-confidence in caring for patients with incurable illness varied. Participants hoped for increasing availability and accessibility of Palliative Care, and extension of clinical education in Palliative Care at all levels (undergraduate, postgraduate, specialization).

**Conclusions:**

Absence of training has resulted in misconceptions and ignorance of common concepts and practices in Palliative Care. Palliative Care education needs to be systematically developed and integrated into clinical training within Armenia. This research may provide a rallying call for changes within the core curricula in Armenia and may also encourage collaborative development in associated countries of the Caucasus region.

**Supplementary Information:**

The online version contains supplementary material available at 10.1186/s12904-022-00938-z.

## Introduction

The demand for Palliative Care is growing due to global demographic changes, including an increasingly aging society and wider prevalence of non-communicable diseases (NCDs); diseases that account for 68% of worldwide deaths [1, 2]. Data from the World Health organization suggests that more than 40 million people each year would benefit from Palliative Care [3], with 78% of these living in low and middle income countries, such as Armenia [1, 4–6].

A lack of undergraduate education and training programs has been identified as major barrier to the development and integration of Palliative Care within existing health care systems [7–10]. Palliative Care education should be considered a core element of health professionals’ training [11, 12], but instead appears to be sporadically integrated, as is the case in Armenia [10, 13].

### Context

Armenia and its healthcare system is confronted with political and economic challenges, including limited funding, a high rate of out-migration (esp. workforce migration) and poverty [14]. The publicly funded “Basic-Benefit-Packet” provides limited universal health coverage, but does not include Palliative Care, excepting the administration of opioids [14, 15]. 80% of Armenia’s health expenditure is privately financed by the population [14, 16].

The current health care provision structures within Armenia impacts on patients with palliative care needs. The majority of patients die at home, with elderly homes/home care limited and/or unaffordable to most. As a result, hospitals often provide long-term care. Within the hospital setting, patients often remain uninformed about their medical condition and information is directed to the family instead [5].

NCDs, especially cardiovascular diseases and cancer, are the primary cause of death in Armenia. Approximately 60–70% of Armenia’s end-of-life patients would benefit from Palliative Care [5, 15]. However, Palliative Care is a fairly new medical field in Armenia with limited implementation, expecting the formation of a Palliative Care task force, integration of few Palliative Care teams and development of “long-term sustainable national strategy for Palliative Care for 2017-2019” [15, 17]. Further, training packages have been developed and delivered, but there remains a lack of specialists, and as yet, no specific Palliative Care unit has opened [10, 15, 17].

Besides limited availability and accessibility to Palliative Care, and a lack of Palliative Care education, associated challenges include an uninformed public, widespread opiophobia and restrictive regulations on access to narcotics that limit clinical availability: annually, only 3% of all patients in need, receive morphine [5, 15, 17, 18].

### Objectives

This research aimed to assess newly qualified physicians’ understanding of Palliative Care, their perception of Palliative Care education in Armenia, as well as their personal experiences in providing such care.

## Method

A constructivist qualitative approach to data collection and analysis was employed, as this aims to provide a general understanding of perceptions, problems and tendencies, and although not intended to be representative, findings may be transferable to other settings [19, 20]. This research was approved by the Ethics Committee of the Uniklinik RWTH Aachen (EK063/16; date: May 2nd, 2016). COREQ (Consolidated criteria for Reporting Qualitative research) and SQUIRE 2.0 (Standards for Quality Improvement Reporting Excellence) checklists were used to guide reporting (Additional File 3, Additional File 4).

### Sample

Based on parallel research in India and China, *n* = 20 was the targeted number of interview participants. Participant inclusion criteria included approximately one year of professional clinical experience, graduation from an Armenian medical school, and employment as a physician in Armenia. Gender and postgraduate specializations were mapped but no stratification was necessary.

### Interview guide

In relation to previous research in China and India, an interview guide (Additional File 1) with open-ended questions was employed. Inspired by the EAPC’s (European Association for Palliative Care) recommendations for an undergraduate Palliative Care curriculum, the interview guide addressed the participants’ knowledge regarding: basics of Palliative Care, pain and symptom management, psychosocial and spiritual aspects, ethical and legal issues, communication, teamwork and self-reflection [21]. In addition, the residents’ perception of their competence, and their expectations of the effects of Palliative Care were queried.

### Data collection

Interviews were conducted in July and September of 2016 in Yerevan. AT, lecturer at Yerevan State Medical University (YSMU), arranged contact with the interviewees and/or their supervisors. The interviews took place during work or at separate appointments. Privacy was intended but not always possible, due to colleagues being present in clinical settings. A participant information leaflet clarified the purpose of the study, procedures, risks, benefits, and confidentiality. The interviewer CH, a female German medical student, performed all interviews in English, partially with the help of translators. The participants knew about the researcher’s profession and interests in the findings. Pre-study interview preparation and training of CH was facilitated by FE. Field notes were taken but not included in the analysis. Findings were not directly returned to the participants and no interview was repeated.

### Analysis

**Fig. 1:**
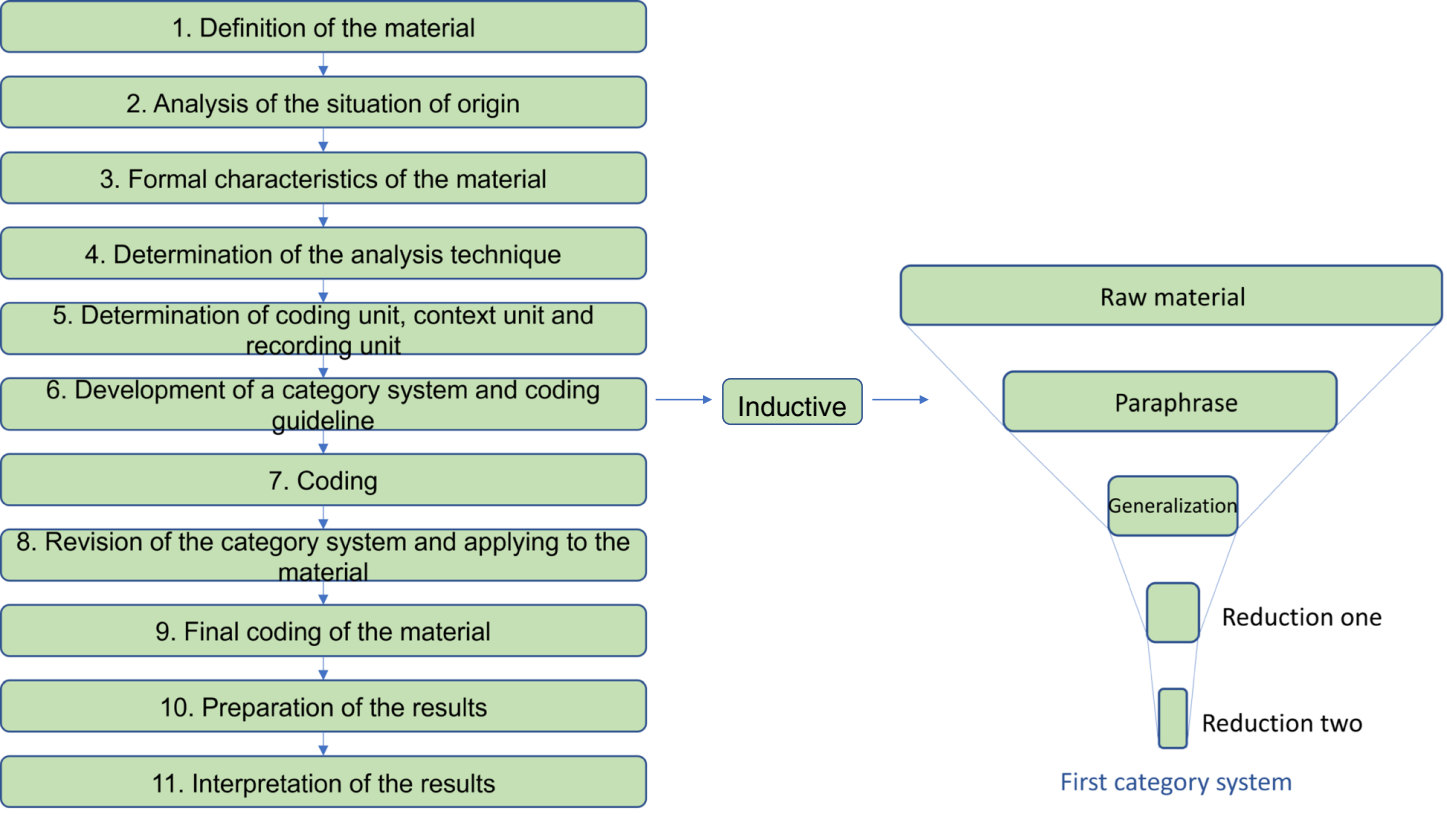
Process of the analysis. Based on: Mayring, P. Qualitation Inhaltsanalyse 2015, pp. 62 ff [20]

All interviews were audiotaped, transcribed, and anonymized with the software f4transkript by CH. To focus the large amount of interview material, Philipp Mayring’s approach of a “summarizing Qualitative Content Analysis” was used [19, 20]. The research material comprised complete transcripts of all interviews (*n* = 20). Fig. 1 illustrates the process of the analysis.

Initially, 25% of the material was used to develop a first category system and coding guideline. To revise this category system, five different interviews were analyzed and coded with the first category system/coding guideline using the software MAXQDA (Version 12). This revised category system, including main-, sub- and sub-sub-categories, was subsequently applied to the whole research material. All information was coded by the interviewer. Key points and significant examples were reworked in discussion with FE. After a first coding, the entire material was reworked a second time, comparable statements were recorded, written, and summarized in the results. Double naming of contents was only counted once.

## Results

The analysis of the data resulted in eight main-categories:

1. Personal medical career
2. Palliative Care (in Armenia)
3. Personal factors and experiences in Palliative Care
4. Palliative Care education
5. Communication
6. Pain and symptom management
7. Multidisciplinary approach
8. Wishes and hopes for Palliative Care in Armenia

Sub-categories were marked in italic. The entire category system can be found in the supplemental material (Additional File 2).

### Sociodemographic characteristics and personal medical career (Table 1)

Twenty medical residents with eleven to thirteen months of work experience participated in the interviews. The respondents worked in eleven different departments and graduated in 2015 from YSMU or the Armenian Medical Institute. The YSMU respondents equated 4.5% of all YSMU graduates in 2015. In total, 15 h and 38 min of interview date were recorded. The average length of the interviews was 47 min (range, 19–74) – four interviews were completely, two partly, translated into English.

**Table 1:** Participants’ sociodemographic and medical career characteristics

Variable	Interviewees (*n* = 20)
Gender (%)	
Female	13 (65%)
Male	7 (35%)
University (%)	
Yerevan State Medical University	18 (90%)
Armenian Medical Institute	2 (10%)
Postgraduate specialization (%)	
Allergology and Rheumatology	1 (5%)
Anesthesiology	2 (10%)
Cardiology	3 (15%)
Dermatology	1 (5%)
General practitioner	1 (5%)
Internal Medicine	2 (10%)
Neurology	4 (20%)
Oncology	1 (5%)
Pediatrics	3 (15%)
Radiology	1 (5%)
General surgery	1 (5%)
Additional working experience (%)	
Ambulance Service	3 (15%)
Master of Public Health	1 (5%)

**Fig. 2:**
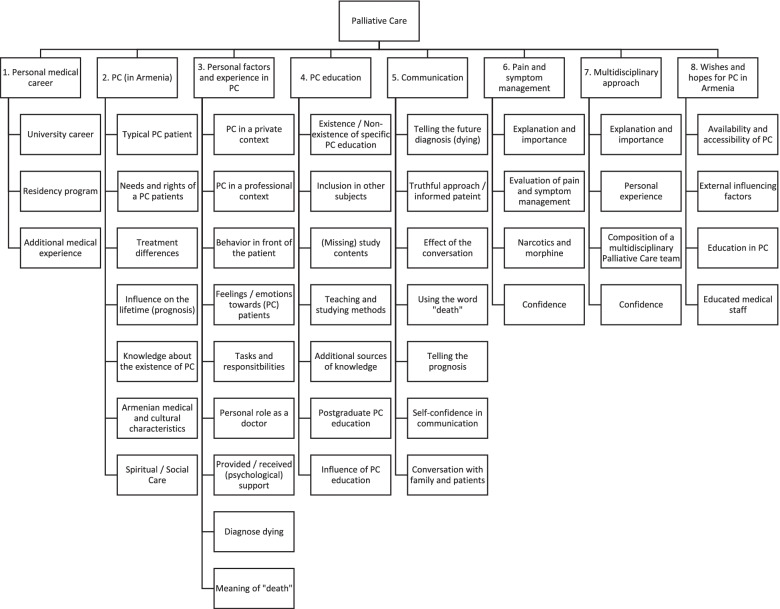
Main- and sub-categories

Figure 2 displays all headings of the main- and sub-categories which are shown in detail in the results. Considering the detailed interview guide, several categories overlap with the interview questions.

### Palliative Care (in Armenia) (Fig. 2)

The interviewees’ responses to the question about the definition and understanding of Palliative Care can be structured in 7 sub-categories (Fig. 2). *Typical Palliative Care patients* were described as those with cancer. Some equated oncological patients and Palliative Care, others described Palliative Care patients with various diseases (e.g. chronic, non-oncological diseases, such as those within cardiology or neurology). Pain was frequently named as the primary symptom, few mentioned other symptoms e.g. emotional stress and bedsores.

Interviewees frequently focused on *Palliative Care patients’ needs and rights* (Table 2). Often named was the importance of managing pain and symptoms. Emotional support and a positive attitude were identified to be helpful to patients.

**Table 2:** Characteristics of the sub-category “Needs and rights of a Palliative Care patient”

Sub-category	Sub-sub-category	Example
Needs and rights of a Palliative Care patient	Support (spiritual/ emotional / psychological / social / accepting diagnosis / daily tasks and activities)	*“Palliative Care means that besides the pills and drugs it needs to be, they should give them also psychological support, rehabilitation support, physiotherapy. Besides the main drug healing.” (Interview 11)*
	Treatment (pain and symptom management / life-supporting)	*“It’s for the people who have no cure, so you just try to alleviate their symptoms and their pain.* “*(Interview 4)*
	Improvement of the quality of life	*“Basically, to put the person in the best position possible and give them comfort in their last days.” (Interview 14)*
	Truthful approach	*“In my opinion being truthful to the patient is the best way to go.” (Interview 2)*

Explained *differences* of Palliative Care from “usual care” included Palliative Care as long-term treatment with death as a result and symptomatic, instead of healing, therapy. A few participants did not make a difference between Palliative Care and Non-Palliative Care patients. All interviewees talked about the *influence on the lifetime (prognosis)*: patients who are in their end-of-life period, suffer from incurable diseases or have a reduced lifetime. Individuals named “prolonging life” as a purpose of Palliative Care (“*We can’t radically treat their disease, so you just have to prolong their life.” (Interview 2))*.

The *knowledge about the existence of Palliative Care in Armenia* was fragmented: some respondents reported no knowledge; others identified hospices, commercial Palliative Care services (home care) and inclusion of Palliative Care in oncology departments (such as Muratsan hospital, Mikaelyan hospital and Malatia medical center). Some highlighted the lack of Palliative Care facilities. A few explicitly described inadequate quality and insufficient development of Palliative Care in Armenia.

Identified *medical and cultural characteristics* included the withholding of information from patients and the important role of family, neighbors, and friends. Some stated that family/friends take over the tasks of psychologists, others identified psychologists as an important group in the care of Palliative Care patients, and considered them an important part of multidisciplinary teams. Caring for end-of-life patients at home was perceived as common, and financial barriers to access healthcare (Palliative Care) were discussed. These characteristics lead to particular challenges for patients without family or friends. Explanations of Spiritual Care equated religion. Part of the group spoke about the existence of Spiritual Care in Armenia, while others denied its existence.

### Personal factors and experiences in Palliative Care (Fig. 2)

Some residents described their *experiences in a private context*: families care for patients at home, lack of open communication with patients, no availability of Palliative Care facilities/home care. In a *professional context* a number of interviewees reported no experience, few mentioned Palliative Care patients during work (e.g. emergency department/ambulance).

Regarding the *behavior in front of patients*, confident appearance was often considered important. Some specified this to be necessary, no matter if they were confident or not. Regarding *feelings and emotions*, opinions varied. Some described avoiding or suppressing emotions at work, others emphasized a need for emotional engagement to facilitate a caring, empathic approach. Many respondents explained difficulties during the care of Palliative Care patients, explicitly emotional difficulties (being stressed, nervous, anxious) or sadness, but also due to the complexity of care. Some excluded Palliative Care as an option for their medical career. Others discussed a neutral, unemotional approach towards Palliative Care patients (Table 3).

**Table 3:** Characteristics of the sub-category “Behavior in front of patients” and “Feelings and emotions towards (Palliative Care) patients”

Sub-category	Sub-sub-category	Example
Behavior in front of patients	Impart confidence	*“But I am trying to look confident. Not about, I’m not sure if I feel like that but I am trying to look confident, so the patient will be more calm.” (Interview 17)* *“If I am saying something seriously, I should say it confident because you know, if you are saying not so confident, if you are not sure that, of your diagnosis it could makes much worser. I am trying to be sure in my diagnosis to do the best to understand the situation and after that I could be confident. But if I am not sure in the diagnosis, of course I am not confidence and I am trying to find other ways of treatment and to help the patient.” (Interview 13)*
	Cold, rational, neutral approach	*“Doctors should be able to judge like the situation and deal with it. Like keep too much emotion out of it.” (Interview 3)* *“I didn’t want to use this word but oncologist, chemotherapists must be coolblooded.” (Interview 12)*
	Behavior similar to a friend	*“I think useful that the patient must feeling that you care about him. […*] *Patient must feeling your care […*]. *Emotion, I think it is very important for patient” (Interview 7)*
	Respectful approach	*“I am trying to be gently and honestly with that patient because they need to use their time that they have to live.” (Interview 13)*
Feelings and emotions towards (Palliative Care) patients	Difficult / hard	*“It probably going to be very hard for me like I might have many sleepless nights […].*” *(Interview 4)*
	Resistance / no contact wished / emotional / sad	*“I feel sad at this time because I am, I think that I am a little bit emotional for doctor […]that’s why it will be better, for me, when I will not take part in the treatment of the oncological and other [Palliative Care] patients […*]” *(Interview 22)* *“I am trying to do my best for that patients because it is my work but if we are speaking honestly for me sometimes it takes so much energy and after that I feel completely empty, that’s why it will be better, for me, when I will not take part in the treatment of the oncological and other [Palliative Care] patients.” (Interview 13)* “*It’s difficult to work with them.” Question: “What is difficult?” “With emotions. With me this is, emotions. With the emotions.” (Interview 11)*
	Neutral	*“I realized that he will die and I don’t think about that. It’s not came me, my emotional status in hospital is stop.” (Interview 9)*
	Nervous / fearful / stressful	*“I don’t feel comfortable because sometimes they ask me and then I am getting so nervous. […] But when they ask me those questions I am just getting nervous and they can read on my face how I am at that time embraced and like thinking how I am going to do that.” (Interview 17)* *“But Palliative Care it’s very traumatic for me.” (Interview 22)* *“When [the patient] entered second time to the hospital and he couldn’t recognize [me] and it was very hard for [me …] Especially it was stressful because [I] knew him, [I] saw him at first when he was very healthy, look very health.” (Interview 6 (translated))* *“So, [I] didn’t went to Oncology center because of that fears.” (Interview 18 (translated))*
	Helpful	*“Palliative Care can help me to treat my patient in high quality.” (Interview 12)*

Most interviewees outlined *their tasks* as being responsible for helping, healing, or caring, including medical treatment (pain and symptoms), giving emotional attention, transferring positive emotions, being a person of trust and more. Some saw their duty in “fighting death”. Since residency programs are seen as education, some residents highlighted *their role* of being students, not doctors, which may lead to the development of different competencies.

Demands for *support* were addressed to supervisors, colleagues, family and friends. Most participants reported no knowledge of, or non-existing, psychological support. Some would appreciate this; others would reject support (particularly psychological) in order to manage situations themselves.

The understanding of *diagnosing dying* needed to be specified by distinguishing between death and dying which was partly related to the language barrier. Individuals described symptoms, appearances and their confidence which was, if existing, related to knowledge and experience. Many respondents described the *meaning of “death”* with a biological understanding, or as the result of a medical event. Frequently, “death” was classified as a part of lifecycle, but was also related to feelings like fear, uncertainty and the question “why”.

### Palliative Care education (Fig. 2)

Independent *Palliative Care undergraduate education and Palliative Care education* were largely reported to be *non-existent*, but education was partly *integrated into other subjects*, e.g. Oncology or Bioethics. Reported *study contents* mainly included disease related topics and pain management. In connection to Palliative Care, psychological, social, and emotional support as well as euthanasia were mentioned by few. Some identified existing education on communication and breaking bad news, others reported the opposite. This was also repeated for education on diagnosing dying. Where engaged, the main *teaching methods* included lectures and seminars, observation of experienced doctors, group discussions and practical education. Besides university education, *additional sources of knowledge* included books, journals, and online resources for self-study.

The majority of interviewees received *no postgraduate Palliative Care education*. Learning from supervisors was a main part of residency education, which partly included training on communication (of breaking bad news). The importance of learning through patient contact was stated by many.

In the absence of specific Palliative Care education programs, reported *influences of education* relate to knowledge from different subjects: some noticed this as influencing on their confidence, abilities, skills and knowledge in Palliative Care. Others mentioned the importance of naming the term Palliative Care within the relevant clinical setting as important and necessary to begin to develop any understanding of Palliative Care practice. Some explained an improvement of the personal approach towards patients with life-limiting diseases due to education. Individuals evaluated practical experiences in patient care and observation of supervisors to be more important than university or residency education.

### Communication (Fig. 2)

*Telling a diagnosis* *(of dying),* was determined not to be part of their job by most. Still, key elements in this process were identified, indicating some clinical development (Table 4).

**Table 4:** Mentioned requirements for telling a diagnosis to a patient

Requirements for telling a diagnosis	
	Certainty of the patients’ future death
	Appropriate surrounding (i.e. quiet atmosphere)
	Preparation of the patient beforehand
	Invitation of company
	Understandable communication of the situation

Difficulties in communication were related to the emotional impact on the resident, and the manner of telling a diagnosis/prognosis to the family instead of the patient; which was explained to be common in Armenia. Reasons for this included personal consent, believe that the truth was too hard for a patient, and fear of trouble with families. A number of interviewees disagreed with this procedure and advocated for an *informed patient*, which led to internal conflicts between common practice and their personal approach.

Described *effects of conversation* with families included difficulties of acceptance, blaming others for the disease and a great commitment for the patient. According to some, bad news leads to patients being traumatized, helpless, stressed, considering suicide and not accepting their situation.

Many supported the avoidance of *using the word “death”*. Some accepted using “death” in a conversation with relatives but not with patients. This avoidance was justified because it was perceived patients would be shocked, lose hope and the respondents would feel personally discomforted.

Only some reported of experience in *telling the prognosis*. Asking for prognosis was described to be common in Armenia. Some participants also reported the challenges of dealing with relatives who demand to withhold a prognosis from the patient. Instead of focusing on a prognosis, alternatives included focusing on the treatment, cure, the patient’s present life, fighting the disease and bringing hope. Some emphasized that consequences and expressions of diseases differ, which makes accurate prognostication incredibly difficult, and thus best avoided. If communicating prognosis, the approaches varied between talking around it, telling percentages of survival rates, explaining the current situation and doctors’ assessing the patients’ condition before telling/not telling a prognosis. Experience, personal approach and intuition would influence the handling of these situations.

No respondent felt completely *confident in communication*. Influencing factors for higher self-confidence included education and knowledge (on diseases, possible therapies, patient related factors), work/personal experiences, increased patient contact, perceived role as a doctor and the sense of doing the right thing/helping and giving hope. A number of interviewees felt more confident in the *conversation with the family than with patients*: families were identified to act tougher and know their relative better than physicians; feelings of patients often challenged the residents. Some described facilitating conversations with patients through their acceptance and coping of their current deteriorating health situation.

### Pain and symptom management (Fig. 2)

The cohort *explained* their personal understanding and perspectives on the *importance of pain and symptom management* for Palliative Care patients, discussing and *evaluating* the particular challenges within Armenia.

Participants wished for wider availability and accessibility to *opioids*, especially access to patient-controlled analgesia (PCA) and oral/transdermal preparations. Most respondents reported no experience with opioids during their personal clinical practice but were aware of administration for oncological, polytraumatic diseases, AIDS, or myocardial infarction. The prescription process was characterized as complicated and strictly regulated due to fear of addiction, loss of control over the patients’ self-administration and the fear of resale. There was disagreement about the possibility of using opioids at home and the availability for certain diseases.

Most interviewees stated *confidence* in prescribing analgesics for Palliative Care and Non-Palliative Care patients. Some felt challenged by both, personal and general difficulties: complex indications for analgesics, limited availability and complex prescription process, and fear of personal failure in treating severe pain.

### Multidisciplinary approach (Fig. 2)

Multidisciplinary teams were understood as teams working together to share knowledge, opinions, procedures and therefore find the best solution for a patient. Both patients and doctors were thought to benefit from the multidisciplinary approach. Many physicians highlighted the need of a psychologist/psychiatrist in *a multidisciplinary Palliative Care team*. Most residents proposed their own profession as a core part of a multidisciplinary team, but several other professions were also mentioned including e.g. emergency care, physiotherapy and nursing. Some described their *experiences* in multidisciplinary teams and noted that this experience increased their *confidence* in Palliative Care.

### Wishes and hopes for Palliative Care in Armenia (Fig. 2)

A general wish for extension of Palliative Care in Armenia was apparent. The cohort demanded increasing *availability and accessibility*: specialized facilities (hospices, hospital departments) and home care; high quality care with psychological, social and rehabilitative support; financial accessibility; expanded availability of pain medication. *External influencing factors* included policy changes, financial support and research. An improvement of *education in Palliative Care* was thought to be crucial, with university education starting in an advanced academic year. Individuals emphasized the need of *educated medical staff* by implementing Palliative Care in postgraduate training, Palliative Care specialization programs and Palliative Care residencies (abroad or in Armenia). Some wished for the expansion of existing Palliative Care education, more intensified practical education with patient contact and access to diverse teaching material. The focus for education should be concentrated on communication (“breaking bad news”), emotional/psychological support of Palliative Care patients/families, pain and symptom management, typical Palliative Care diseases and related problems, diagnosing dying and evidence-based approaches to support dying patients. Individuals wished for parallel education of the Armenian population.

## Discussion

The residents in this study echo the wider perception from the literature that Armenia lacks adequate availability and accessibility of Palliative Care, and specifically Palliative Care education [10, 15]. The existing educational gap may have led to misconceptions of the function of Palliative Care, impacted upon confidence in supporting patients with Palliative Care needs and limited the support available to patients.

The wide range of understanding Palliative Care suggests a missing structured education for clinicians: beyond pain, symptoms common in patients with advanced disease were not frequently explained (gastrointestinal symptoms, nausea, pulmonary symptoms, etc.). Misconceptions included “Palliative Care equals oncological care” or that the “goal of Palliative Care is to prolong life”, indicating a lack of experience, understanding and potentially supportive skills/abilities.

The limited knowledge on the existence and aims of Palliative Care matched the limited availability and development of Palliative Care services [15, 17]. Identified characteristics of care within Armenia, such as the majority of care occurring at home, uninformed patients, importance of the family and limited accessibility (financial barriers) of Palliative Care corresponded with former research [5, 15, 18]. Varying understanding of Spiritual Care (“Spiritual care equals religion”) and Social Care might indicate a wider lack of conceptual understanding of the aims and principles of Palliative Care.

Individual approaches towards end-of-life patients and complexities regarding emotional involvement and difficulties were prevalent. Interestingly, these challenges are those amenable to being addressed by education, clarifying concepts of physicians’ tasks in end-of-life care. Previous research highlights the importance of practical and autonomous work for the improvement of capabilities in working with Palliative Care patients [11]. Residents frequently identified a need for knowledge and experience to increase self-confidence. However, being seen as “students” during residency might lead to perceived diminished responsibility and independence. Since observation was a main studying method, changes in skills (e.g. communication skills) would be likely to take longer since supervisors being observed are unlikely to have had significant Palliative Care education and may not be appropriate role models in all areas of care.

A lack of structured education may also explain the varying, non-standardized approaches regarding the explanation of diagnosis and prognosis. *No resident* expressed great confidence in their communication, even though the interviewees’ preferred to appear confident. The fear of distressing consequences due to breaking bad news and inner conflicts between withholding information (culturally wanted) but personally wanting to address the patients’ questions, led to interviewees’ discomfort and contrasts with widespread opinions of the need to facilitate patients’ autonomy [22, 23]. Education should contain knowledge on ethical concerns about communication and patients’ autonomy. Little education on approaching a Palliative Care patient, was indeed contrary to needed psychological support and structured communication [10, 21, 24].

Availability and accessibility of opioids remains one of the major challenges when caring of patients with incurable diseases in Armenia. The participants’ descriptions of these barriers and perceptions echo literature [5, 18, 25].

The interviewees’ hopes and wishes expressed their dissatisfaction with the state of Palliative Care in Armenia, highlighting needed changes and challenges. Addressing this, a multipronged strategy including undergraduate education, postgraduate education as well as specialization training needs to be implemented [11, 12]. To facilitate this, the support of policy makers, complemented by national and international funding, is required [6].

Palliative Care is most needed in low- and middle-income countries such as Armenia with only little access for patients in need [1, 6]. In a regional context, Armenia remains behind other countries in terms of its development with no specific Palliative Care education at all and a lack of specialized care for patients with incurable illnesses [10, 25]. In comparison, neighbors such as Georgia provides some specific education at all levels (undergraduate, postgraduate, specialization) as well as some Palliative Care facilities [17, 26, 27]. These examples may be taken into consideration to implement this important part of patient care in Armenia as well.

Although this study focuses on Armenia, it may be that patterns of misconceptions, individual approaches, as well as limited confidence in the care of Palliative Care are applicable to countries with a similar status comparable historical background, particularly Eastern Europe and the Caucasus region [8, 10].

### Limitation of the study and methodical critique

This analysis provided descriptive, non-representative statements and identified opinions of young doctors in Armenia. All data was conducted in 2016 – and information given, referred to the health care/political/educational system in and before 2016.

Limitations of the study included a convenience sample, and the sample may have inherent bias through selection by supervisors who were keen to support the research. Due to the presence of translators or colleagues, the openness and relationship between interviewer and respondent might have been affected. However, translators also improved the residents’ ability to express themselves in their mother tongue, as compared to English. Partly, English conducted interviews lacked comprehensible and understandable terms (Spiritual Care, death/dying) and needed explanation that may have influenced the impartiality of the doctors. Previous questions regarding education (communication, pain, etc.) might have affected answers regarding wishes/hopes for Palliative Care.

Nevertheless, the semi-structured approach secured relevant content and the opportunity to engage a wide range of opinions. Continuous consultations were held with AT to interpret cultural differences. Limitations regarding the analysis included the use of only one coder.

## Conclusion

This research presents a wide range of information on residents’ knowledge, understanding and confidence in Palliative Care, and Palliative Care education in Armenia. This analysis sets a base for further exploration and contributes to the world-wide assessment of the need of Palliative Care education, particularly undergraduate education. Palliative Care education in all levels should be introduced in Armenia, and a nationwide study about confidence of residents who received Palliative Care education could follow, to examine the effects of training. This research may be used in negotiations with policy makers and may provide a basis for the further development of a Palliative Care curriculum in Armenia. Results may also be applicable to Palliative Care Education in other countries of the Caucasus region and beyond.

## Supplementary Information

**Additional File 1.** “Interview Guide Palliative Care and Palliative Care education in Armenia”. Final version of the interview guide used in the interviews in Armenia.

**Additional File 2.** “Category-system Palliative Care and Palliative Care education in Armenia”. Final version of the complete category-system with Main categories, sub-categories and sub-sub-categories as well as typical examples for the whole research.

**Additional File 3.** “COREQ (Consolidated Criteria for Reporting Qualitative Studies)-Checklist for the manuscript “Improvable Education in Palliative Care A qualitative research about Palliative Care education from the perspective of young physicians”. A second tool to revise the quality of the manuscript.

**Additional File 4.** “SQUIRE 2.0 (Standards for QUality Improvement Reporting Excellence): revised publication guidelines from a detailed consensus process”. A tool to revise the quality of qualitative research and explanation for application of standards of qualitative research in this study.

## Data Availability

All data is stored at the Department of Palliative Medicine of RWTH Aachen University as well as partially with Dr. med. Carolin Hagedorn (carolin.hagedorn@rwth-aachen.de). The datasets used and analyzed are not publicly available due to protection of participant’s statements and identities but are partially available from the corresponding author on reasonable request.

## Abbreviations

EAPC: European Association for Palliative Care
NCD: Non-communicable diseases
YSMU: Yerevan State Medical University
