# Supplementary material for: Palliative Care education in Armenia: perspectives of first-year Armenian physician residents
Source: BMC Palliat Care. 2022 Apr 20;21:53. doi: 10.1186/s12904-022-00938-z (PMC9019978; doi:10.1186/s12904-022-00938-z)
Supplement: Supplementary file 1 — Additional File 1. “Interview Guide Palliative Care and Palliative Care education in Armenia”. Final version of the interview guide used in the interviews in Armenia. [file 12904_2022_938_MOESM1_ESM.pdf]

Additional File 1: Interview Guide Palliative Care and Palliative Care education in Armenia

1. **Could you please give me a brief summary of your professional development?**
2. **Please could you describe your personal understanding of palliative care?**
3. **Could you describe your experience of education in palliative care?**
4. **Could you tell us about how you experience the quality of the education in palliative care?** →Do you feel more prepared for the care of incurable and dying patients and their families after the education?
5. **How confident and able do you feel to**
    - deal with difficult questions
    - tell patients / families that they
        - cannot be cured / are dying?
6. **How confident and able do you feel to provide Pain and Symptom Management in a palliative situation?**
7. **How confident and able do you feel to diagnose dying?**

Is it common to tell the diagnosis or prognosis to the patient / the family?

8. **Working in a Multidisciplinary Team** → What does it exactly mean to you?
9. **Could you describe your attitudes towards death and dying?**
    - As a Health Care Professional
    - As an individual
10. **Could you describe the personal support from the faculty you did receive?**
11. **What is your idea of your role as a physician?**
12. **What are your hopes regarding future palliative care in Yerevan or Armenia?**

Thank you very much for your time and for sharing your thoughts with me!
