# Supplementary material for: Palliative Care education in Armenia: perspectives of first-year Armenian physician residents
Source: BMC Palliat Care. 2022 Apr 20;21:53. doi: 10.1186/s12904-022-00938-z (PMC9019978; doi:10.1186/s12904-022-00938-z)
Supplement: Supplementary file 2 — Additional File 2. “Category-system Palliative Care and Palliative Care education in Armenia”. Final version of the complete category-system with Main categories, sub-categories and sub-sub-categories as well as typical examples for the whole research. [file 12904_2022_938_MOESM2_ESM.pdf]

Additional File 2: Category-system Palliative Care and Palliative Care education in Armenia

Main category	Subcategory	Sub-sub-category	Typical example
**Personal medical career**	University career	- attended university (YSMU, Armenian Medical Institute)<br>- duration of education<br>- additional education	*"My university is Yerevan State Medical University after M. Heratsi" Interview 19*
	Residency program	- description of current postgraduate position	*"I am resident in Neurology department." Interview 14*
	Additional medical experience	- further education/trainings	*"Three years I worked as a parametic and now here work as a doctor, physician." Interview 21*
**Palliative Care (in Armenia)**	Typical Palliative Care patient	- cancer<br>- chronic, non-oncological diseases<br>- symptoms (pain/bedsores/emotional instability)<br>- patients' characteristics (strong/individuality/not supported)	*"Cancer patients, neurological patients, lots of fields." Interview 2*<br>*"Patients who have pain, physical or emotional." Interview 15*
	Needs and rights of a Palliative Care patient	- support (spiritual/emotional/psychological/social/accepting diagnosis/daily tasks and activities)<br>- treatment (pain and symptom management/life-supporting)<br>- improvement of the quality of life<br>- truthful approach towards the patient	*"It's for the people who have no cure, so you just try to alleviate their symptoms and their pain." Interview 4*<br>*"Palliative Care means (...) it means to be near the patient, near, I mean not only physical near. Near the patient in the difficult moment." Interview 1*

	Treatment differences	- PC treatment (long-time care/symptomatic treatment/positive handling/emotionality)<br>- no treatment differences<br>- different questions from patients<br>- death as a result	*"I think that Palliative Care needs to have more attention and concern to the patient."* Interview 11<br>*"I am a doctor and I mustn't difference between two patients. I think that it is not right, it's wrong." Interview 5*<br>*"Because if we are patient with oncological disease the patient needs of drugs are for pain management, for management of some other symptomatic problems, that's why if we are just imagining this kind of patient are in that moment in the ICU [Intensive Care Unit] or in other department he will also take the same medication as in Palliative Care that's why I guess that there is no so big difference." Interview 13*
	Influence on the lifetime	- life-limitation<br>- end-of-life period<br>- incurability<br>- prolong life	*"By saying Palliative Care I first of all understand that they are the terminal patients that are probably already not curable." Interview 17*
	Knowledge about the existence of Palliative Care in Armenia	- no knowledge of existence<br>- no department<br>- no PC residency/no specialists<br>- hospices<br>- commercial PC<br>- inclusion in Oncology department<br>- quality of PC	*"Palliative Care? I don't know, here or, yes or no. I don't know." Interview 11*<br>*"I knew some places it is called hospice. Have you heard about it? I don't know. Actually, there are patient with oncological diseases and why not. I guess it is a kind of Palliative Care." Interview 13*<br>*"I believe there are a few hospices but not really available for everyone as far as I know." Interview 2*

	Armenian medical and cultural characteristics	- role of the family, friends, neighbors<br>- role of psychologists<br>- home care and hospital care<br>- economic situation<br>- out-of-country treatment	*“Our patients, their problems are telling on his relatives, neighbors, and our patients relatives are his psychologists. Neighbors are psychologists. Everyone is listening about this problem.” Interview 3 (partially translated)*<br>*“Actually, he was at home, I mean the mostly patients, they are at home, not in the hospitals.” Interview 13*
	Spiritual / Social Care	- no knowledge about spiritual or social care<br>- (non-) existence of spiritual or social care<br>- importance of spiritual and social care<br>- meaning of Christianity	*“In official no. The priest must, will come in our clinic and talk with every patient. If the patient’s family want, they invite.” Interview 11*
**Personal factors and experience in Palliative Care**	Palliative Care in a private context	- no experience<br>- family members/friends	*“My aunt's husband was die after cancer and this palliative treatment was a very big problem. We didn't have.“ Interview 3 (partially translated)*
	Palliative Care in a professional context	- no experience<br>- patients in own department<br>- patients during paid employment	*“I have a small experience with patients from our ambulance because people who have cancer call ambulance a lot for analgetics.” Interview 21*
	Behavior in front of the patient	- impart confidence<br>- cold, rational, neutral approach<br>- behavior similar to a friend<br>- respectful approach<br>- hypothetical idea	*“But I am trying to look confident. Not about, I'm not sure if I feel like that but I am trying to look confident, so the patient will be more calm.” Interview 17*<br>*“I didn't want to use this word but oncologist, chemotherapists must be coolblooded.” Interview 12*<br>*“I think useful that the patient must feeling that you care about him. […] Patient must feeling your care […]. Emotion, I think it is very important for patient.” Interview 7*

	Feelings / emotions towards (Palliative Care) patients	- difficult/hard<br>- resistance/no contact wished - emotional/sad<br>- neutral<br>- stressful<br>- nervous/fearful<br>- helpful	*"I feel sad at this time because I am, I think that I am a little bit emotional for doctor. Because of that I don't, I don't choose therapy, I choose diagnostic radiology for don't contact with patients very close. Because it's little trauma for me when I see... ." Interview 22*<br>*"It's difficult to work with them. [What is difficult?] With emotions. With me this is, emotions. With the emotions." Interview 11*
	Tasks and responsibilities for the patient	- emotional and social support<br>- transfer positive emotions<br>- proper pain management<br>- support relatives<br>- fight death<br>- explain situation and create understanding for disease	*"Attention, caring and treatment." Interview 14 (partly translated)*<br>*"So for her, Palliative Care means that besides the pills and drugs it needs to be, they should give them also psychological support, rehabilitation support, physiotherapy. Besides the main drug healing." Interview 11*<br>*"So, [I] didn't went to Oncology center because of that fears." Interview 18 (translated)*
	Personal role as a doctor	- help and cure the patient<br>- still being a student<br>- specific competencies during residency<br>- contact for medical information<br>- person of trust	*"So as a doctor [I see myself] as, to work more for the patients, work a lot for the patients to make their life more healthy but for chronical patients [I], which has the death end, [I see myself] to make their life more easy during that period. To make them easy and work a lot with them." Interview 16 (translated)*<br>*"Like, saving lives, helping people (laughing)." Interview 17*<br>*"So, she is saying right now she doesn't have any experience because she is not actually working as a specialist, she is just a resident." Interview 4*

	Provided / received (psychological) support	- from different departments<br>- from family and friends<br>- from colleagues<br>- from superior doctors<br>- not available<br>- (not) wanted (psychological) support	*"I have a colleague, a lot of colleagues but not with everyone here. I can speak with my colleagues, my colleague, she is very close for me. Maybe with her and my mom." Interview 5*<br>*"[I] just will keep [my] emotions, feeling inside and [I] will not ask for help for someone." Interview 18 (translated)*
	Diagnosing dying	- dying = consequence of diseases<br>- symptomatic description<br>- description of appearance<br>- (non-) existent confidence	*"How do you diagnose dying? You don't diagnose dying, you just diagnose the problem which could lead to death." Interview 2*<br>*"His look, his all habitus, say he, he has a poor prognosis. When you say you, when you see patient, his eyes, you can probably thinking about it. But he has a big, he has a, he has a pain syndromes, he has a problem with intestines, always diarrhea, and even he went, even when he didn't die next day, several days ago." Interview 12*
	Meaning of "death"	- biological understanding<br>- part of life<br>- releasing emotions<br>- (non-) existing differences between personal and professional meaning	*"So for, as a doctor it is that stopping heart, stopping breathing but as a person (...) So for her it is the circle of life. That's how she see the word death as a person but as a doctor it's stop breathing, heart is stopping." Interview 16 (translated)*<br>*"For me. I fright of my relatives' deaths. Death is very bad. It's end of life. It's how this person were and now he is not. I afraid maybe more others death than mine." Interview 10*
**Palliative Care education**	Existence / Non-existence of specific Palliative Care education	- non-existence of specific PC education - existence of specific PC education	*"We don't have a course for palliative care and I think just resident of oncology learn that for a long time, maybe months, I don't know how long, but in university, in our plan we don't have palliative care. […] Maybe there is any course, but not in my plan." Interview 15*

	Inclusion in other subjects	- in Bioethics<br>- in Family Medicine<br>- in Internal Medicine<br>- in Oncology<br>- in Pharmacology<br>- in Psychology<br>- in Rehabilitation and Physiotherapy<br>- in Social Health<br>- in Surgery	*"We have one course in palliative care. It was when we was five course (5th year) It was in family medicine."* Interview 3 (partially translated)<br>*"Maybe oncology more or less."* Interview 21<br>*"We only learn about palliative medicine in other courses. In surgeon, surgeon patients about, therapy patients about palliative. This kind."* Interview 3 (partially translated)
	(Missing) study contents	- disease related topics<br>- pain management<br>- psychological, emotional or social support<br>- general care/approach of PC patients<br>- euthanasia<br>- (no) communication<br>- (no) telling diagnosis<br>- (no) diagnosis dying<br>- no memory of contents	*"No. I mean we have, when we are taught the disease, we are taught it from the beginning. Like the etiology, the pathology, the complications and of course in the complications, death is included in the complications kind of. And, but, that's the only way we talk about it."* Interview 17<br>*"[…] they taught us to be careful about how to tell the patient, whether you should or you should actually tell the relatives. Should the patient be aware of the situation?"* Interview 4 (translated)
	Teaching and studying methods	- observation<br>- reading<br>- discussion<br>- interactive teaching<br>- (missing) practical education	*"It was something like practice during that classes. They met patients and doctor choose how to behave, what to ask, what to do?"* Interview 6 (translated)<br>*"Only learn in paper what was written. We didn't have experience with patients, we have now [in residency] experience some, after graduate this university."* Interview 3 (partially translated)

	Additional sources of knowledge	- brochures, journals<br>- books<br>- online<br>- doctors<br>- experience<br>- conferences	*"We get lot of magazines, new magazines with modern trial, modern chemotherapy drugs, chemotherapy trials and even about palliative care issues." Interview 12*<br>*"Everything is from, I think, from my ambulance cause when we have analgesics." Interview 21*
	Postgraduate Palliative Care education	- (non-) existence<br>- study contents<br>- teaching methods<br>- patient contact/own experience	*"First days we go with our tutors to see the patients and we saw and heard how she, our tutor, communicate with patients and after that we make our experience ourselves." Interview 15*
	Influence of (Palliative Care) education	- no influence on abilities, skills, knowledge<br>- on approaching a (Palliative Care) patient<br>- on communication<br>- on skills and abilities<br>- on knowledge<br>- on personal distance	*"No, I think before university I didn't know about it anything. But now maybe I know very small information, but I have that little bit." Interview 19*<br>*"I can't say [if I feel more prepared caring for incurable patients after the university education] because as I said. We were just, it was just lectures. We would learn it on paper, we wouldn't have any experience. […] Until the moment you actually start working with patients one on one, we would actually learn how to deal with them - for palliative." Interview 2*
**Communication**	Telling the future diagnosis (dying)	- (non-existent) experience<br>- self-assessed confidence<br>- description of setting	*"[And do you feel confident to tell the diagnosis to the patient?] Yeah. Maybe 70%." Interview 15*<br>*"I can't say for sure cause I have never been in this kind of situation." Interview 21*

	Truthful approach / informed patient	- informing family first<br>- family assessing patient’s condition<br>- family asks for uninformed patient<br>- informing patient first<br>- importance of informing the patient<br>- personal conflict<br>- personal assessment of patient’s condition<br>- partly telling diagnosis	*“In Armenia very often relatives didn't want to, didn't want that patient know when he has a cancer and that's why in that patients we very often speaking with relatives.”* Interview 12<br>*“I don't know, I think we try... I can't explain. (...) We think that patient will be more happy without know about his true diagnose. I think so. [And do you tell the family the truth?] Yeah. Most of the situations we tell the parents. The family but not the patient.”* Interview 5<br>*“In my opinion being truthful to the patient is the best way to go. You can't just sugarcoat it and say "No, you're gonna be okay". You have to just be truthful to them, let them know what is waiting for them.”* Interview 2
	Effect of the conversation	- (non) acceptance by the family/the patient<br>- reaction of the family (blame/behavior changes)<br>-devasted/traumatized/sad/ depressed patient<br>- hopeful patient	*“The family, they are usually in denials so they try to blame everyone and everything else - including the doctor.”* Interview 4<br>*“He thinks that a patient that is going to die and has incurable disease somehow has a little bit hope that he will cure.”* Interview 6
	Using the word “death”	- (not) using the word “death”<br>- different addressees<br>- description of the conversation<br>- individuality	*“Usually no, I prefer to - we don't know when patient will die, this know only god and we say your diagnose it not so much good. This kind. I don't prefer to say "you will die".”* Interview 3 (partially translated)<br>*“I think I must use that word. […] Because it's a step of our life and everybody must know about death and in the end of our life we all must die and it's normal word for me.”* Interview 5

	Telling the prognosis	- frequent question<br>- different questioners<br>- reserved approach<br>- relation to patient’s condition<br>- description of telling a prognosis<br>- exact prognosis can’t be given<br>- (no) experience	*“People do want to know the prognosis. Even the simplest problems, simple neurosis, they want to know their prognosis. And it is better to let them know [their prognosis] rather than let the patient go and search the internet and find wrong information.” Interview 4 (translated)*<br>*“The doctor has due to the cultural norms, has to be very careful and [attentive] on how to tell the prognosis.” Interview 3 (partially translated)*<br>*“I say that "I don't know [how long you can live]". I don't... It can know only god. Doctors cannot know.” Interview 5*
	Self-confidence in communication	- work experience/patient contact<br>- education<br>- knowledge<br>- certainty of diagnosis/prognosis - position as a doctor<br>- personal characteristics/life-experiences<br>- feeling of helping/giving hope<br>- enough timing<br>- full explanation of situation<br>- age, emotional state, disease of the patient	*“I think I can learn it in years, not for first year or second. Maybe after ten years ago I will be confident, but not now.“*<br>*“He can't say that "very confident" but also he can't say that "I don't feel any confident". It depends on patients age, problem, disease.”*<br>*“Because when you go to a clinic you see how the doctor interacts, communicates with different patients […] And later you just, that's the way to have those skills and the communication is better.”*
	Conversation with family and patients	- easier conversation with the family<br>- easier conversation with the patient<br>- description of common procedures in the conversation with the family/the patient	*“I don't know. Telling, telling, telling a person that he will die is much more difficult for me than telling relative, for me. I think that is very personal.” Interview 21*<br>*“Because maybe the relatives know him better, the patient better and they could explain him about his diagnosis more right and they could know who tell him that he listen about his diagnosis more, not tragically.” Interview 15*

**Pain and symptom management**	Explanation and Importance	- importance<br>- different types/understanding of pain<br>- difficulties<br>- individual perception	*“Pain it's a feeling that, for me, that person, that disturb person and it, it's a different feeling for each one. I think it's high grade of any feeling - it's a high grade of any feeling.” Interview 5*
	Evaluation of pain and symptom management	- non-availability<br>- missing understanding	*“In Armenia sometimes it is difficult to get the certain drugs because maybe part, some of the family don't know about it what kind of pills and drugs need to be used for the patient.” Interview 18 (translated)*
	Narcotics and morphine	- usage/personal experience<br>- application<br>- (home) availability<br>- accessibility<br>- fears	*“If they are not gonna give it in big batches, let's say for a month, and the patient has to go every two days to receive the medications - she is saying there should a service for, let's say the policlinic, to deliver the medication. She is also saying: maybe it's not a possible to give patients here in big batches because they like, in a couple of days the patient would decide "oh the pain is too much, I'm gonna take more" so to keep the control they have to keep it in small batches but maybe a delivery system would be better.” Interview 4 (translated)*
	Confidence	- (non) existing confidence<br>- influencing factors	*“He is saying you can't be always confident about this drug issue because different patients respond different.” Interview 3 (partially translated)*
**Multidisciplinary approach**	Explanation and Importance	- (no) knowledge<br>- benefits for patient<br>- impotence<br>- tasks	*“This specialists must working together to treat one patient. “ Interview 12*<br>*“For her it is important to have that multidisciplinary, multiprofessional team.” Interview 16*
	Personal experience	- no experience<br>- in residency<br>- personal effect	*“[And have you worked in a multidisciplinary team?] No, I didn't work. “ Interview 3 (partially translated)*

	Composition of a multidisciplinary Palliative Care team	- named specializations	*"For example, in Oncology sphere, in Oncology in multidisciplinary team it will consist with doctor, oncologists, medical oncologists, surgery, medical surgery, pathology, diagnose and psychology, psychosocial worker and of course in multidisciplinary team Palliative Care workers also is working - I think this specialists must working together to treat one patient." Interview 12*
**Wishes and hopes for Palliative Care in Armenia**	Availability and accessibility of Palliative Care	- available for everyone<br>- PC facilities<br>- imagination of possible realization of PC (quality care/multidisciplinary teams/psychological and social support/provided pharmaceuticals/financial availability)	*"In a hundred years we don't have Palliative Care in our country and I don't have hope. [Why?] Because people who must do this work, they are busy how get money. How get money and go holidays - Italia, Francia, they don't care about people who need Palliative Care in my opinion." Interview 7*<br>*"I think the most times in near years I think we will have a high level service of Palliative Care because in this time there are some doctors, they are working in this sphere, they are in contact with foreign specialists, with Germany, Austria, Russia and I think most times because this stuff very important part of our treatment, it's very useful and very... it can be help to overcome disease, cancer disease especially." Interview 12*
	External influencing factors	- governmental (+ financial) support<br>- evaluation and research	*"After that our government will give some attentive for this and our government should be give some finance to - financially some big problem." Interview 3 (partially translated)*<br>*"Enough good evaluation of the palliative care, because right now, we have very big problems with that, we need it." Interview 13*

Education in Palliative Care	- general demand<br>- timing<br>- addressees of education<br>- educational time<br>- teaching methods<br>- study contents	*"Yeah, I would like to [have more education on Palliative Care], because every specialist, every doctor could meet patient who needs Palliative Care." Interview 15*<br>*"It will good if we know more about the patients, about their emotions, about how we can help them, what we do to help them." Interview 11*
Educated medical staff	- specialization program/residency<br>- education abroad	*"I hope that, at least in Oncology center or the residents in Oncology should, could study for long term Palliative Care." Interview 15*
