# Supplementary material for: Palliative Care education in Armenia: perspectives of first-year Armenian physician residents
Source: BMC Palliat Care. 2022 Apr 20;21:53. doi: 10.1186/s12904-022-00938-z (PMC9019978; doi:10.1186/s12904-022-00938-z)
Supplement: Supplementary file 3 — Additional File 3. “COREQ (Consolidated Criteria for Reporting Qualitative Studies)-Checklist for the manuscript “Improvable Education in Palliative Care A qualitative research about Palliative Care education from the perspective of young physicians”. A second tool to revise the quality of the manuscript. [file 12904_2022_938_MOESM3_ESM.pdf]

Additional File 3: COREQ (Consolidated Criteria for Reporting Qualitative Studies)-Checklist for the manuscript "Improvable Education in Palliative Care A qualitative research about Palliative Care education from the perspective of young physicians"

Allison Tong, Peter Sainsbury, Jonathan Craig, Consolidated criteria for reporting qualitative research (COREQ): a 32-item checklist for interviews and focus groups, *International Journal for Quality in Health Care*, Volume 19, Issue 6, December 2007, Pages 349–357, https://doi.org/10.1093/intqhc/mzm042

No	Item	Requested Information	Reference in Manuscript
**Domain 1: Research team and Reflexivity**			
Personal Characteristics			
1.	Interviewer	Carolin Hagedorn, corresponding author	Methods: Data collection, page 6
2.	Credentials	None	Title page, page 1
3.	Occupation	Medical student	Methods: Data collection, page 6
4.	Gender	Female	Methods: Data collection, page 6
5.	Experience and training	Individual training and regular consultation by FE. Theoretical knowledge acquisition of the practical implementation in advance by CH.	Methods: Data collection, page 6
Relationship with participants			
6.	Relationship established	No special relationship between participants and interviewer. AT contacted the interviewees or their supervisors who were visited during their working hours. The ones who were busy, were contacted afterwards via telephone or messenger to set up a separate appointment.	Methods: Data collection, page 5-6
7.	Participant knowledge of the interviewer	Interviewees were informed about CH being a medical student from Germany who is interested in the field of Palliative Care and its	Methods: Data collection, page 6.

		implementation in Palliative Care.	
8.	Interviewer characteristics	Referring to previous research and descriptions of AT, the researcher expected a lacking implementation of Palliative Care education. Difficulties in contacting participants, cultural barriers and language problems were expected.	Description not included in the manuscript.
**Domain 2: study design**			
Theoretical framework			
9.	Methodological orientation and Theory	The method of summarizing qualitative content analysis referring to Mayring was chosen. Explanation of the concrete application and development of the category system.	Methods: Analysis, page 6, 7 and Figure 1
Participant selection			
10.	Sampling	Description of inclusion criteria.	Methods: Sample, page 5-6
11.	Method of approach	Participants were contacted by AT or their supervisors. CH then visited the participants and either interviewed right away or made an appointment. Beforehand the informed consent was read, explained and signed.	Methods: Analysis, page 6, 7 and Figure 1
12.	Sample size	n = 20	Results: Sociodemographic characteristics and personal medical career, page 7-8
13.	Non-participation	n = 1	Results: Sample, page 5. Sociodemographic characteristics and personal medical career, page 8

Setting			
14.	Setting of data collection	Quit room was intended but not always given.	Data collection, page 6. Discussion: Limitation of the study and methodical critique, page 18
15.	Presence of non-participants	Sometimes. Translators or colleagues.	Data collection, page 6. Discussion: Limitation of the study and methodical critique, page 18
16.	Description of sample	Sociodemographic characteristics and personal medical career, Table 1	Results: Sample, page 5. Sociodemographic characteristics and personal medical career, page 8
Data collection			
17.	Interview guide	Interview guide can be found in a separate file	Additional file 1: “Interview Guide Palliative Care and Palliative Care education in Armenia”
18.	Repeat interviews	None	Methods: Data collection, page 6
19.	Audio/visual recording	Audio recording	Methods: Analysis, page 6
20.	Field notes	Were taken but not included in the analysis.	Methods: Data collection, page 6
21.	Duration	19-74 minutes, average length 47 minutes	Results, page 9
22.	Data saturation	A completeness or representative results cannot be given by the interviewed number of participants. However, tendencies and findings clearly identify problems and relevant subjects.	Method: page 5 Discussion: Limitation of the study and methodical critique, page 18
23.	Transcripts returned	None	Methods: Data collection, page 6
**Domain 3: analysis and findings**			
Data analysis			
24.	Number of data coders	One coder, CH with frequent consultations of FE	Methods: Analysis, page 7

25.	Description of the coding tree	Naming of 8 main categories, detailed description of the focused 5 categories in Table 2 and complete Category-System Additional file 4	Results: page 7. Results: Figure 2 Additional File 2: "Category-system Palliative Care and Palliative Care education in Armenia"
26.	Derivation of themes	Interview guide conducted in relation to EAPC's recommendation. Themes and Coding-Tree was derived from data.	Method: Interview guide, page 6. Results: page 7. Figure 2. Additional File 4: "Category-system Palliative Care and Palliative Care education in Armenia"
27.	Software	MAXQDA and f4 transkript	Methods: Analysis, page 6-7
28.	Participant checking	Not applicable since transcripts and findings were not returned.	Methods: Data collection, page 6
Reporting			
29.	Quotations presented	Applicable	Results: Palliative Care (in Armenia), Personal factors and experiences in Palliative Care. Table 2 and 3, page 9-10 and 25-26. Additional File 2: "Category-system Palliative Care and Palliative Care education in Armenia"
30.	Data and findings consistent	Applicable	Results: Category 2-8. Page 9-16. Figure 1, Table 2 and 3, page 9-10 and 25-26. Additional File 2: "Category-system Palliative Care and Palliative Care education in Armenia"
31.	Clarity of major themes	Applicable	Results: Category 2-8. Page 9-16.

			Figure 1, Table 2 and 3, page 9-10 and 25-26.<br>Additional File 2: “Category-system Palliative Care and Palliative Care education in Armenia”
32.	Clarity of minor themes	Applicable	Results: Category 2-8. Page 9-16.<br>Figure 1, Table 2 and 3, page 9-10 and 25-26.<br>Additional File 2: “Category-system Palliative Care and Palliative Care education in Armenia”
