# Supplementary material for: Palliative Care education in Armenia: perspectives of first-year Armenian physician residents
Source: BMC Palliat Care. 2022 Apr 20;21:53. doi: 10.1186/s12904-022-00938-z (PMC9019978; doi:10.1186/s12904-022-00938-z)
Supplement: Supplementary file 4 — Additional File 4. “SQUIRE 2.0 (Standards for QUality Improvement Reporting Excellence): revised publication guidelines from a detailed consensus process”. A tool to revise the quality of qualitative research and explanation for application of standards of qualitative research in this study. [file 12904_2022_938_MOESM4_ESM.pdf]

Additional File 4: SQUIRE 2.0 (Standards for QUality Improvement Reporting Excellence): revised publication guidelines from a detailed consensus process

Ogrinc G, Davies L, Goodman D, Batalden P, Davidoff F, Stevens D. SQUIRE 2.0 (Standards for QUality Improvement Reporting Excellence): revised publication guidelines from a detailed consensus process. BMJ Qual Saf. 2016 Dec;25(12):986-992. doi: 10.1136/bmjqs-2015-004411. Epub 2015 Sep 14. PMID: 26369893; PMCID: PMC5256233.

**Title and Abstract**	
**1. Title**	The title includes the main results and the research methods. It indicates the need of improvable education in Palliative Care in Armenia and the key factor of Armenian respondents' perception of this specific field.
**2. Abstract**	The abstract is subdivided in background, methods, results and conclusions and presents keywords for the research at its end. Therefore, all key information is summarized.<br>It provides results to provide information to aid in searching and indexing.
**Introduction and Background**	
**3. Problem Description**	Introduction and background explain the need of Palliative Care education as well as the lack of it in Armenia. Moreover it is mentioned that no data exists on the perception of education or Palliative Care status in Armenia.
**4. Available knowledge**	Introduction, context and objectives broadly describe the need of Palliative Care, lack of it in Armenia, insufficient undergraduate education which is needed to improve provided care. It also explains the applicability to other countries and gives references for statements.
**5. Rationale**	By not having similar research for Armenia, models or concepts are not applicable.
**6. Specific aims**	Aim of the study was to assess the perception of newly qualified physicians' understanding of Palliative Care as well as the perception of Palliative Care education in Armenia.
**Methods**	
**7. Context**	The methods describe an orientation on recommendations of EAPC (European Association for Palliative Care) and therefore the development of the interview guide. The setting and timing are presented in the methods and are discussed in the limitations of the study.

**8. Intervention**	Included description of setting, timing, interviewer, and analyzer of the material. A broad description of the research method is given. The tasks of the researcher are presented.
**9. Study of the Intervention(s)**	Not applicable.
**10. Measures**	Description of the chosen method “summarizing qualitative content analysis” and explanation of use.<br>A completeness or representative results are not given by the interviewed number of participants. Explanation for this is presented in the limitations of the study.<br>Descriptions of the methods include data collection methods, data collection instruments (software’s), the number of participants and length of interviews. Techniques to enhance trustworthiness comprise the revision of the category process by a second researcher.
**11. Analysis**	Qualitative approach as a semi-structured interview study, analyzed in a summarizing qualitative content analysis referring to Mayring. Explanation of Mayring’s qualitative content analysis and its application.
**12. Ethical Considerations**	Ethical committee, Ethical Approvement as well as the date of the approvement are presented.
**Results**	
**13. Results**	The results are subdivided in main findings and main categories. Quotes and category system can be found in the tables. The results comprise detailed information about the participants and their statements.
**Discussion**	
**14. Summary**	Key statements are summarized in the beginning of the discussion. It states the applicability to other countries with similar existing provision of Palliative Care or comparable cultural background.
**15. Interpretation**	The discussion provides a comparison of the findings with further existing publication but also redefines new findings due to non-existing research beforehand. Influencing context factors were described as well.
**16. Limitations**	Limitations include limitations in the interview situation, randomization as well as analysis process.
**17. Conclusions**	The conclusion gives introduces main findings and gives suggested next steps. Furthermore, it demands for more research, intensified development of Palliative Care and Palliative Care education. It allows to use the data for further argumentation.
**Other information**	
**18. Funding**	No funding for this research. No conflict of interest in this study.
